# Diagnostic efficacy of circular RNAs as noninvasive, liquid biopsy biomarkers for early detection of gastric cancer

**DOI:** 10.1186/s12943-022-01527-7

**Published:** 2022-02-09

**Authors:** Souvick Roy, Mitsuro Kanda, Sachiyo Nomura, Zhongxu Zhu, Yuji Toiyama, Akinobu Taketomi, James Goldenring, Hideo Baba, Yasuhiro Kodera, Ajay Goel

**Affiliations:** 1 Department of Molecular Diagnostics and Experimental Therapeutics, Beckman Research Institute of City of Hope, 1218 S. Fifth Avenue, Monrovia, CA 91016 USA; 2 Department of Gastroenterological Surgery, Nagoya University Graduate School of Medicine, Nagoya, Japan; 3 Department of Gastrointestinal Surgery, Graduate School of Medicine, The University of Tokyo, Tokyo, Japan; 4 Department of Biomedical Sciences, City University of Hong Kong, Hong Kong SAR, China; 5 Department of Gastrointestinal and Pediatric Surgery, Division of Reparative Medicine, Institute of Life Sciences, Mie University Graduate School of Medicine, Tsu, Mie Japan; 6 Department of Gastroenterological Surgery I, Graduate School of Medicine, Hokkaido University, Sapporo, Hokkaido Japan; 7 Section of Surgical Sciences, Department of Cell and Developmental Biology, Epithelial Biology Center, Vanderbilt University School of Medicine, Nashville VA Medical Center, Nashville, TN USA; 8 Department of Gastroenterological Surgery, Graduate School of Medical Sciences, Kumamoto University, Kumamoto, Japan; 9 Department of Surgery, Japanese Community Health Care Organization Kumamoto General Hospital, Kumamoto, Japan; 10 The International Research Center for Medicine Sciences, Kumamoto University, Kumamoto, Japan; 11 City of Hope Comprehensive Cancer Center, Duarte, CA USA

**Keywords:** Circular RNAs, Gastric cancer, Biomarker panel, Non-invasive liquid-biopsy assay

## Abstract

**Background:**

Majority of gastric cancers (GC) are diagnosed at advanced stages which contributes towards their poor prognosis. In view of this clinical challenge, identification of non-invasive biomarker for early diagnosis is imperative. Herein, we aimed to develop a non-invasive, liquid-biopsy based assay by using circular RNAs (circRNAs) as molecular biomarkers for early detection of GC.

**Methods:**

We performed systematic biomarker discovery and validation of the candidate circRNAs in matched tissue specimens of GC and adjacent normal mucosa. Next, we translated the discovered circRNA based biomarker panel into serum samples in a training and validation cohort of GC patients (*n* = 194) and non-disease controls (*n* = 94) and evaluated their diagnostic performance. In addition, we measured the expression of circRNAs in serum samples of pre- and post-surgical GC patients and evaluated the specificity of circRNAs biomarker panel with respect to other gastro-intestinal (GI) malignancies.

**Results:**

We identified 10-circRNAs in the discovery phase with subsequent validation in a pilot cohort of GC tissue specimens. Using a training cohort of patients, we developed an 8-circRNA based risk-prediction model for the diagnosis of GC. We observed that our biomarker panel robustly discriminated GC patients from non-disease controls with an AUC of 0.87 in the training, and AUC of 0.83 in the validation cohort. Notably, the biomarker panel could robustly identify even early-stage GC patients, regardless of their tumor histology (diffuse vs. intestinal). The decreased expression of circRNAs in post-surgery serum specimens indicated their tumor-specificity and their potential source of origin in the systemic circulation.

**Conclusions:**

We identified a panel of 8-circRNAs as non-invasive, liquid-biopsy biomarkers which might serve as potential diagnostic biomarkers for the early detection of GC.

**Supplementary Information:**

The online version contains supplementary material available at 10.1186/s12943-022-01527-7.

## Introduction

Gastric cancer (GC) is the fifth most-commonly diagnosed cancer, and as per GLOBOCAN 2020 reports, this malignancy ranks as the fourth leading cause of cancer-related deaths worldwide [1]. It was reported that in the United States, almost one third of patients with GC are diagnosed at an advanced stage with distant metastasis leading to an overall poor prognosis [2]. Endoscopy remains the gold standard for screening and diagnosing patients with GC. However, the invasive nature of endoscopy results in patient discomfort and can sometimes lead to serious complications [3, 4]. Nonetheless, in a few East Asian countries, endoscopy is available at an affordable cost and has allowed establishment of nationwide endoscopic surveillance programs. These GC screening programs have been clinically significant, as they have facilitated early detection of GC, and have allowed timely treatment interventions which has resulted in an overall improved prognosis for this malignancy [5, 6]. Noteworthily, the conventional serological tumor markers such as carcinoembryonic antigen (CEA), carbohydrate antigen (CA) 19–9 and CA 72–4 are inadequate for detecting GC due to their poor sensitivity and specificity [6]. The lack of availability of clinically actionable and noninvasive screening modalities remains one of the critical barriers for improving the early detection of GC, highlighting the need to develop noninvasive, affordable and robust biomarkers that can be implemented globally for the timely and early detection of gastric neoplasia.

Research efforts in this regard in the recent years aimed at the development of such noninvasive biomarkers have mostly been based on measuring the expression of various circulating noncoding RNAs (ncRNAs), primarily microRNAs (miRNAs) and long noncoding RNAs [7–15]. However, in more recent years, emerging evidence indicates that another class of ncRNAs, defined as circular RNAs (circRNAs), might actually offer a highly, an even more robust cancer detection [16], including GC. The circRNAs are single-stranded ncRNAs that are produced from pre-mRNAs through transcriptional back-splicing [17]. Based upon their functional relevance, it seems that they primarily act as ‘miRNA sponges’ which allows them to control the expression of various growth-regulatory genes that are downstream targets of various miRNAs [17–19]. Intriguingly, just in the past few years, several circRNAs have been identified that play a pivotal role in the pathogenesis of multiple human cancers including GC [20, 21]. From a clinical biomarker perspectives, these have garnered increasing attention because of their inherent circular configuration, which makes them resistant to RNase-mediated degradation and allows them to be easily detectable in a variety of body fluids with a high degree of tissue specificity [22, 23] – which are some of the ideal and much needed characteristics required for the development of clinically robust biomarkers.

However, the current landscape for the development of circRNA-based diagnostic biomarkers is still young and suffers from various shortcomings, including the use of non-comprehensive biomarker discovery approaches, the lack of translation of the tissue-based discovered biomarkers into blood (serum or plasma), and the failure to validate biomarkers in independent cohorts of clinical specimens [24]. In order to address these important gaps in knowledge and shortcomings of existing literature, in the present study we conducted a systematic and comprehensive biomarker discovery approach, followed by the validation and development of these biomarkers as a noninvasive, liquid biopsy-based assay for the early detection of GC. Accordingly, we were successfully established an 8-circRNA biomarker panel that not only enables early detection of patients with GC but was significantly specific and superior than currently used classic tumor markers for the early detection of gastric neoplasia.

## Methods

### Study design and patient cohorts

The overall workflow for this study is illustrated in Supplementary Fig. 1. The study comprised of a systematic and comprehensive biomarker discovery, followed by multiple validation phases. For the biomarker discovery phase, we analyzed circRNA expression profiling data from two independent datasets (GSE89143 and GSE83521). All expression profiling data were downloaded from the Gene Expression Omnibus (GEO) database. Given the small number of patients used in each dataset, we combined these data together for the biomarker discovery efforts. Following biomarker discovery, we interrogated the expression of candidate circRNAs in a pilot cohort of 28 matched GC and adjacent normal mucosal (ANM) tissues by real-time quantitative polymerase chain reaction (RT-qPCR). These specimens were obtained from patients enrolled at Kumamoto University, Japan, between 2008 and 2009. The clinicopathological details of the cohorts are presented in Supplementary Table 1.

For the translation of tissue-based biomarkers into a liquid biopsy-based assay, serum samples from two retrospective cohorts of patients with GC along with non-disease controls were analyzed. The training cohort consisted of 92 patients with GC and 46 endoscopically negative patients (non-disease controls) enrolled at the Kumamoto University, Japan, between 2010 and 2015. The second validation cohort consisted of serum specimens from 102 GC patients and 48 non-disease control subjects enrolled at the Mie University Graduate School of Medicine, Japan, between 2006 and 2017. The clinicopathological details of the cohorts are presented in Supplementary Table 2. In addition, we also analyzed an independent cohort of 24 GC patients, who were enrolled prospectively, from whom we obtained serum specimens prior to curative surgery and 3-month post-surgery. All of these prospectively recruited patients were enrolled during March 2017–March 2018 at the Nagoya University, Japan.

Finally, in order to evaluate the specificity of candidate circRNAs for GC, we compared the performance of our biomarker panel with other gastrointestinal (GI) cancers such as colorectal cancer (CRC), esophageal squamous cell carcinoma (ESCC), pancreatic ductal adenocarcinoma (PDAC) and hepatocellular cancer (HCC). For these analysis, the expression of the final circRNA panel was examined in serum specimens from 20 patients with each of the GI cancers by RT-qPCR. For CRC and ESCC, serum specimens were collected from patients enrolled at the Mie University Graduate School of Medicine, Japan, between 2014 and 2016. For PDAC, serum specimens were collected from patients enrolled at the Nagoya University, Japan, between 2012 and 2014 and for HCC serum specimens were collected from patient enrolled at the Hokkaido University, Japan, between 2008 and 2009.

### Discovery of candidate circRNAs using genomewide circRNA expression profiling datasets

In the discovery phase we first analyzed circRNA expression profiling data using two different GEO datasets to identify candidate circRNAs associated with GC. Among them, GSE89143 contained circRNA expression data from 3 patient matched GC and ANM tissues, and GSE83521 which comprised of circRNA expression profiling from 6 patient matched GC and ANM tissues. First, we performed principal component analysis (PCA) plot analysis of the two datasets and observed one outlier specimen which was excluded from further analysis. Next, we used ComBat parametric adjustment to remove any batch effects within the microarray datasets and performed differential expression analysis using the limma package in R [25]. The candidate circRNAs were initially selected based on the following criteria of log_2_-fold change of > 1 and Benjamini Hochberg-adjusted *p* < 0.05. However, to improve our probability of successful validation of the biomarkers, we prioritized circRNA candidates with a log_2_-fold change > 2; hence using more stringent criteria for selecting candidates for subsequent validation.

### RNA extraction and circRNA expression analysis

Total RNA was isolated from fresh frozen surgical tumor tissues, adjacent normal mucosa and 200 μl of serum specimens by using RNeasy mini kit (Qiagen, Hilden, Germany) and miRNeasy Serum/Plasma Kit (Qiagen) respectively. cDNA synthesis was performed using the High-Capacity cDNA Reverse Transcription Kit (Applied Biosystems, Foster City, CA). The divergent primers for candidate circRNAs were designed by using CircInteractome database [26]. The expression of circRNAs was evaluated by RT-qPCR assays. The RT-qPCR assays were performed using the SensiFAST™ SYBR® Lo-ROX Kit (Bioline, UK) on the Quantstudio 7 Real-Time PCR System (Applied Biosystems). The relative expression levels of target circRNA was normalized against β-actin and fold change was calculated using the 2^-ΔCT^ method. The fold change values were log transformed for further analysis [27]. The sequences of the primers are presented in Supplementary Table 3. In order to confirm the specificity of divergent primers and stability of circRNAs, we have used a two-pronged approach. We not only used a primer design that used splice-site junctions, but also used RNase R treatment to rule out any amplification of linear RNAs. First, total RNA was isolated from AGS gastric cancer cells using the RNeasy mini kit (Qiagen). Thereafter, 2 μg of total RNA was incubated for 15 min at 37 °C in presence or absence of 5 U/μg RNase R (Epicenter Technologies, Madison, WI, USA). The resulting RNA was purified using RNeasy MinElute Cleanup Kit (Qiagen) and the circRNA expression levels were measured by RT-qPCR.

### Statistical analysis

All statistical analyses were performed using the R version 4.0.3, MedCalc Statistical Software version 20.009 (MedCalc Software Ltd., Ostend, Belgium) and GraphPad Prism 8 software (La Jolla, CA). Differential circRNA expression profiling was performed through the limma package in R and the resulting *p* values were adjusted using the Benjamini Hochberg’s method. The logistic regression analysis was also performed using Medcalc. Area under the curve values (AUCs) derived from the receiver operating characteristic (ROC) curves were calculated with CIs using the pROC package in R and ROC curves were compared using DeLong tests in the pROC package. All ROC curves presented in our results are shown with 95% CI. The CI was calculated by 2000 bootstrap replicates. The optimal cutoff points for ROC curves were determined using Youden’s index in the pROC package. The sensitivity, specificity, positive predictive value (PPV), negative predictive value (NPV), precision and accuracy of circRNAs based biomarker panel were calculated across all the cohorts using the report ROC package. Kruskal-Wallis test was performed to assess the statistical significance between early stage, late stage and non-disease controls based on their risk score. A paired t test (one-sided) was used to compare gene expression levels between serum samples collected pre- and post-surgery. A *p* < 0.05 or less was considered as statistically significant.

## Results

### Genome-wide transcriptional profiling identifies circRNAs as candidate biomarkers to distinguish GC from adjacent normal mucosal tissue specimens

**Fig. 1:**
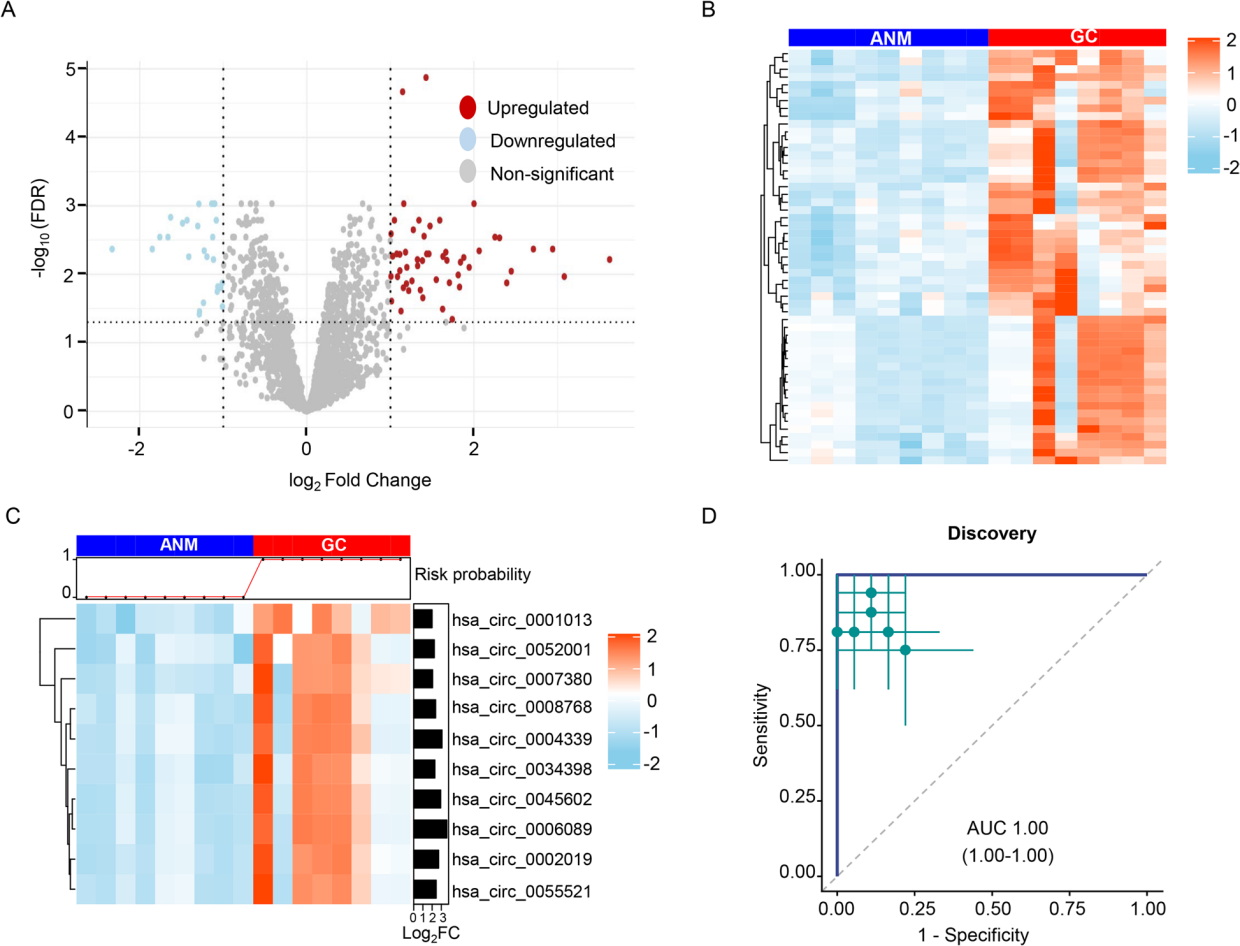
Genome-wide discovery of circRNA candidates for the diagnosis of patients with GC by analyzing transcriptomic expression profiling datasets. **A** Volcano plot illustrates the significantly up- (red) and down-regulated (blue) circRNAs based on log_2_FC > 1 and adjusted *p* < 0.05 derived from the GSE89143 and GSE83521 datasets. **B** Heatmap of upregulated circRNAs between GC patients and matched adjacent normal mucosa (ANM). **C** Heatmap of 10 circRNA candidates based on their log_2_FC > 2 and adjusted *p* < 0.05 between GC patients and adjacent normal mucosa. **D** Assessment of performance of 10 circRNA based biomarker panel by receiver operating characteristic (ROC) curve analysis. ROC curves are shown with 95% confidence intervals (Cis). Green lines in right panel indicate 95% CIs of sensitivity and specificity for each circRNA; green points, optimal threshold for sensitivity and specificity

First, we undertook a systematic and comprehensive analysis to identify circRNA biomarker candidates by analyzing two genome-wide circRNA expression profiling datasets from patients with GC. Based on differential expression analysis, we identified 53 upregulated and 26 downregulated circRNAs (log_2_FC > 1 and adjusted *p* value < 0.05), which are illustrated as a volcano plot in Fig. 1A. Considering that we sought to develop a blood-based non-invasive assay, we prioritized circRNAs that were over-expressed in GC tissues vs. adjacent normal mucosa (ANM), and represent these as a heatmap in Fig. 1B. In addition, in order to develop a clinically feasible assay with a high sensitivity for diagnosing GC, we further increased the stringency for biomarker prioritization. Accordingly, we selected circRNAs that were more robustly expressed in the GC vs. ANM tissues and exhibited a log_2_FC > 2, which led to the identification of 10 candidate circRNAs (Fig. 1C). Next, we performed ROC analysis to evaluate the performance of these circRNA biomarkers as a combination panel for its ability to discriminate GC tissues from ANM. These analysis revealed that this biomarker panel possessed robust diagnostic potential and exhibited an AUC of 1.00 (95% Confidence interval (CI):1.00–1.00, *p* < 0.001; Fig. 1D); hence, highlighting its remarkable diagnostic potential for the identification of patients with gastric neoplasia.

### Validation of the circRNA biomarker panel for its discriminatory potential in GC vs. adjacent normal mucosal tissues

**Fig. 2:**
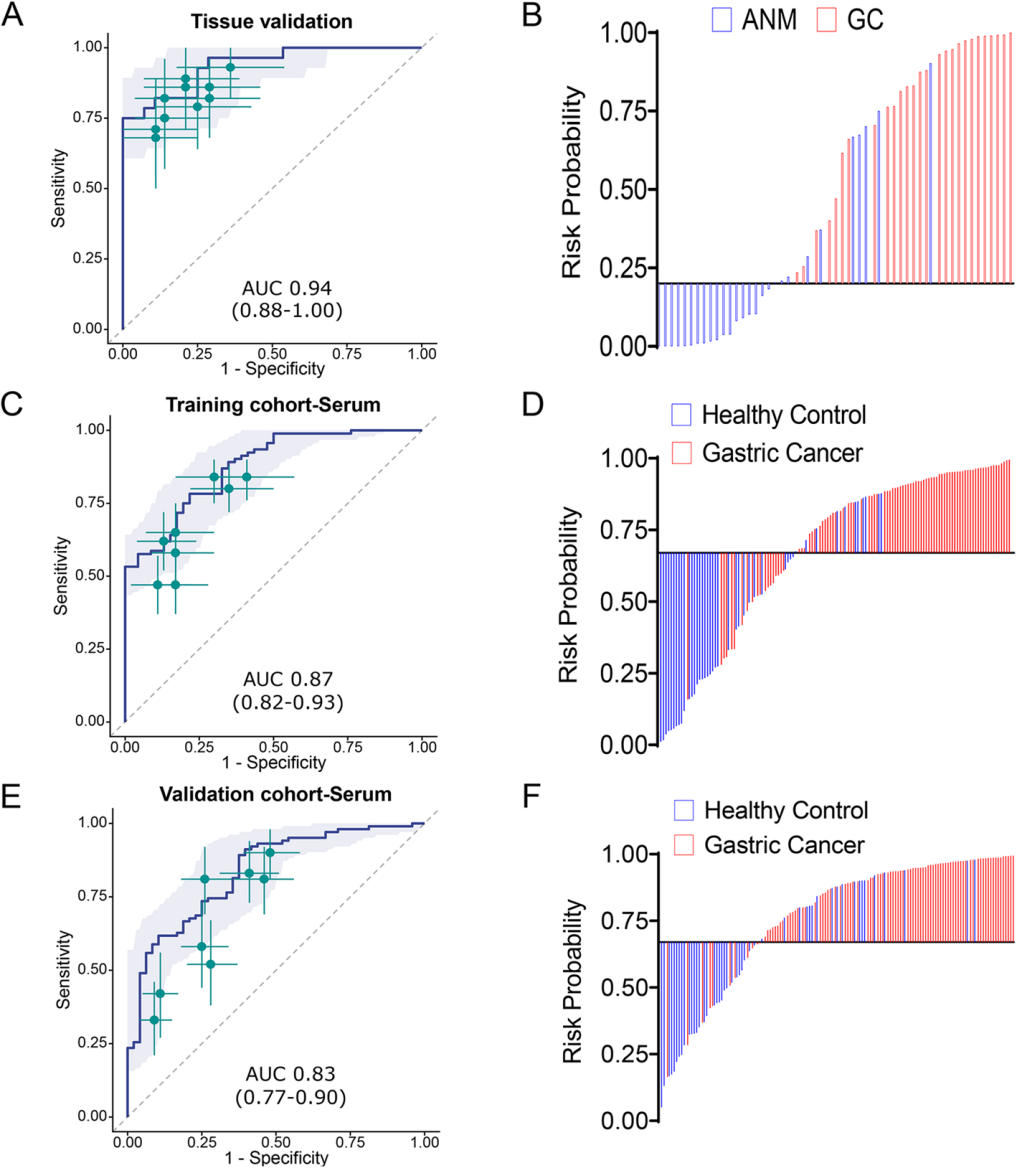
Validation and performance evaluation of circRNA biomarker panel in clinical cohorts of tissue and serum specimens. **A** ROC curve analysis to evaluate the performance of 10 circRNAs based biomarker panel and **B** risk probability distribution plot in a pilot cohort of matched GC tissue specimens and adjacent normal mucosa (ANM). **C** ROC curve analysis to examine the performance of 8 circRNA-biomarker panel and **D** risk probability distribution plot in a training cohort of serum samples from GC patients and non-disease controls. **E** ROC curve analysis and **F** risk probability distribution plots in a serum validation cohort of serum samples from GC patients and non-disease controls. ROC curves are shown with 95% CIs. Green lines in right panel indicate 95% CIs of sensitivity and specificity for each circRNA; green points, best threshold for sensitivity and specificity

Prior to the validation of the identified candidate circRNAs, we ensured the specificity of our assays to specifically amplify circRNAs by performing RT-qPCR assays on the RNA templates that underwent RNAse-R digestion. In these experiments, we observed that all 10 candidate circRNAs were resistant to RNase-R digestion and were successfully amplified by the circRNA-specific divergent primers. Next, we evaluated the performance of 10 circRNA biomarker panel in a tissue-based pilot cohort comprising of 28 tissue specimens from GC patients along with their corresponding ANM by RT-qPCR. The results were further examined by ROC curve analysis to determine the diagnostic power of the biomarker panel individually and in combination for their ability to discriminate GC from ANM. The logistic regression analysis revealed that while individual circRNAs were quite robust, the diagnostic performance of the circRNA biomarker panel was significantly superior and yielded an AUC value of 0.94 (95% CI:0.88–1.00, Sensitivity 75%, Specificity 100% and *p* < 0.001; Fig. 2A and Supplementary Table 4). We further calculated the risk probability based on coefficients of individual circRNAs and the constant derived from logistic regression model as follows: [(2.11568 X hsa_circ_0045602) + (1.67753 X hsa_circ_0008768) + (0.60306 X hsa_circ_0004339) + (− 1.25341 X hsa_circ_0007380) + (2.00083 X hsa_circ_0002019) + (0.93924 X hsa_circ_0055521) + (− 0.49699 X hsa_circ_0006089) + (− 0.076325 X hsa_circ_0034398) + (− 0.65303 X hsa_circ_0052001) + (− 0.30216 X hsa_circ_0001013) + 11.24948. Subsequently, we performed the waterfall plot analysis by dichotomizing the patients based on Youden’s index derived cutoff value of risk probability (Fig. 2B); which once again confirmed that the 10 circRNA panel was quite robust in discriminating between GC vs. ANM tissues.

### Successful translation of tissue-based circRNA panel into a liquid-biopsy based assay in a training cohort of gastric cancer patients

The primary objective of our study underlies the development of a liquid-biopsy based assay for the early-detection of patients with GC. In this regard, we next interrogated the feasibility of translating our tissue based circRNA biomarker panel into a liquid-biopsy based assay by measuring their expression in serum specimens obtained from a training cohort of 92 patients with GC and 46 non-disease controls. We observed that two circRNAs, hsa_circ_0055521 and hsa_circ_0004339, did not express in serum specimens and hence we excluded them from further analysis. We thereafter determined the expression levels of the remaining 8 circRNAs by RT-qPCR assays and analyzed their diagnostic potential by developing a logistic regression model. We observed that 8-circRNA biomarker panel distinguished patients with GC from non-disease control subjects, with an AUC value of 0.87 (95% CI: 0.82–0.93, sensitivity 78.3%, specificity 78.3%, *p* < 0.001; Fig. 2C and Supplementary Table 5). In order to check the robustness of individual circRNAs to diagnose GC we also performed univariate analysis. It was observed that 6 of the 8 circRNAs exhibited a significant association in discriminating GC, while the other two had a *p* < 0.1 (Supplementary Table 6).

Next, we calculated the risk probability based on coefficients for individual circRNAs which were derived from the logistic regression model as follows: [(0.86593 X hsa_circ_0045602) + (0.77243 X hsa_circ_0008768) + (0.37127 X hsa_circ_0007380) + (0.47991 X hsa_circ_0002019) + (− 0.099920 X hsa_circ_0006089) + (− 0.010309 X hsa_circ_0034398) + (− 0.095420 X hsa_circ_0052001) + (0.65834 X hsa_circ_0001013) + 8.27655]. In addition, we also calculated other diagnostic indicators for the performance of this biomarker panel in the serum training cohort, which included sensitivity, specificity, PPV, NPV and accuracy in discriminating GC patients from relevant controls (Table 1). It was observed that liquid biopsy based circRNA biomarker panel exhibited sensitivity of 0.78, specificity of 0.78, accuracy of 0.78 in order to discriminate GC patients from non-disease controls. Based upon this regression model, all GC cases were further dichotomized based on the Youden’s index derived cutoff value of the risk probability and performed waterfall plot analysis to demonstrate the performance of 8-circRNA panel to distinguish GC patients from non-disease controls (Fig. 2D). These results demonstrated the 8-circRNAs based biomarker panel can successfully discriminate GC patients from non-disease controls.

**Table 1:** Summary of diagnostic performance of circRNA-based biomarker panel in discovery, tissue validation, serum training and validation cohorts

Phase	Analysis	Cohort	Control	Cancer	AUC (95% CI)	Accuracy^a^	PPV^a^	Sensitivity^a^	Specificity^a^	NPV
Tissue phase		Discovery cohort	9	8	1 (1.00–1.00)	1 (1.00–1.00)	1 (1.00–1.00)	1 (1.00–1.00)	1 (1.00–1.00)	1 (1.00–1.00)
		Validation cohort	28	28	0.94 (0.88–1.00)	0.88 (0.79–0.95)	1 (1.00–1.00)	0.75 (0.57–0.89)	1 (1.00–1.00)	0.8 (0.70–0.90)
Serum phase	Training	All GC vs controls	46	92	0.87 (0.82–0.93)	0.78 (0.71–0.85)	0.88 (0.82–0.94)	0.78 (0.70–0.87)	0.78 (0.65–0.89)	0.64 (0.55–0.75)
		Early- stage GC vs controls	46	68	0.87 (0.80–0.93)	0.77 (0.69–0.84)	0.84 (0.77–0.92)	0.76 (0.66–0.87)	0.78 (0.65–0.89)	0.69 (0.60–0.80)
		Diffuse GC vs controls	46	45	0.85 (0.77–0.92)	0.75 (0.66–0.84)	0.69 (0.61–0.78)	0.89 (0.80–0.98)	0.61 (0.48–0.76)	0.85 (0.74–0.96)
		Intestinal GC vs controls	46	47	0.9 (0.84–0.96)	0.82 (0.74–0.89)	0.82 (0.73–0.91)	0.83 (0.70–0.94)	0.8 (0.70–0.91)	0.83 (0.73–0.93)
	Validation	All GC vs controls	48	102	0.83 (0.77–0.90)	0.81 (0.75–0.87)	0.83 (0.79–0.89)	0.89 (0.83–0.95)	0.62 (0.48–0.75)	0.73 (0.62–0.85)
		Early- stage GC vs controls	48	69	0.82 (0.74–0.89)	0.78 (0.71–0.85)	0.77 (0.70–0.83)	0.9 (0.83–0.96)	0.6 (0.46–0.73)	0.81 (0.69–0.92)
		Diffuse GC vs controls	48	49	0.84 (0.77–0.92)	0.78 (0.71–0.86)	0.91 (0.82–1.00)	0.63 (0.49–0.78)	0.94 (0.85–1.00)	0.71 (0.64–0.80)
		Intestinal GC vs controls	48	53	0.83 (0.75–0.91)	0.78 (0.70–0.85)	0.73 (0.67–0.81)	0.92 (0.85–0.98)	0.62 (0.50–0.75)	0.89 (0.78–0.97)

*GC* Gastric Cancer, *CI* Confidence interval, *PPV* Positive predictive value, *NPV* Negative predictive value

^a^The value is the average of 2000 bootstrap replicates

### The liquid-biopsy based circRNA panel robustly identifies patients with GC in an independent validation cohort

Following development of the 8-circRNA based risk prediction formula in the training cohort, we further validated its diagnostic performance in serum specimens obtained from an independent cohort of 102 GC patients and 48 non-disease controls. In this case, we calculated risk probability by using the coefficients of each individual circRNA and constant obtained from the logistic regression model established in the serum training cohort. Consistent with our data from the training cohort, the 8-circRNA biomarker panel yet again performed remarkably well and robustly distinguished patients with GC from controls with an impressive AUC of 0.83 (95% CI 0.77–0.90), Sensitivity of 0.89, specificity of 0.62 and accuracy of 0.81 (Fig. 2E, Table 1 and Supplementary Table 7). These result was further confirmed by using waterfall plot analysis by dichomatizing the entire cohort based on Youden’s index derived cutoff value of the risk probability (Fig. 2F).

To further assess the clinical significance of our biomarker panel, we next carried out decision curve analysis (DCA) and calibration plots. The DCA revealed that our circRNA based biomarker panel achieved a significantly higher net benefit than the conventional strategy of treating all the patients or none of the patients across most ranges (Supplementary Fig. 2A). Likewise, the calibration plots were in agreement with regards to the observed vs. predicted probability of treatment (Supplementary Fig. 2B). While a slight overestimation for diagnosis of GC was observed when the predicted probabilities were high (> 0.8), overall our results highlighted that our 8-circRNA panel fared remarkably well in its ability to identify patients with GC.

### The non-invasive circRNA panel robustly identifies patients with the earliest stages of gastric cancer

**Fig. 3:**
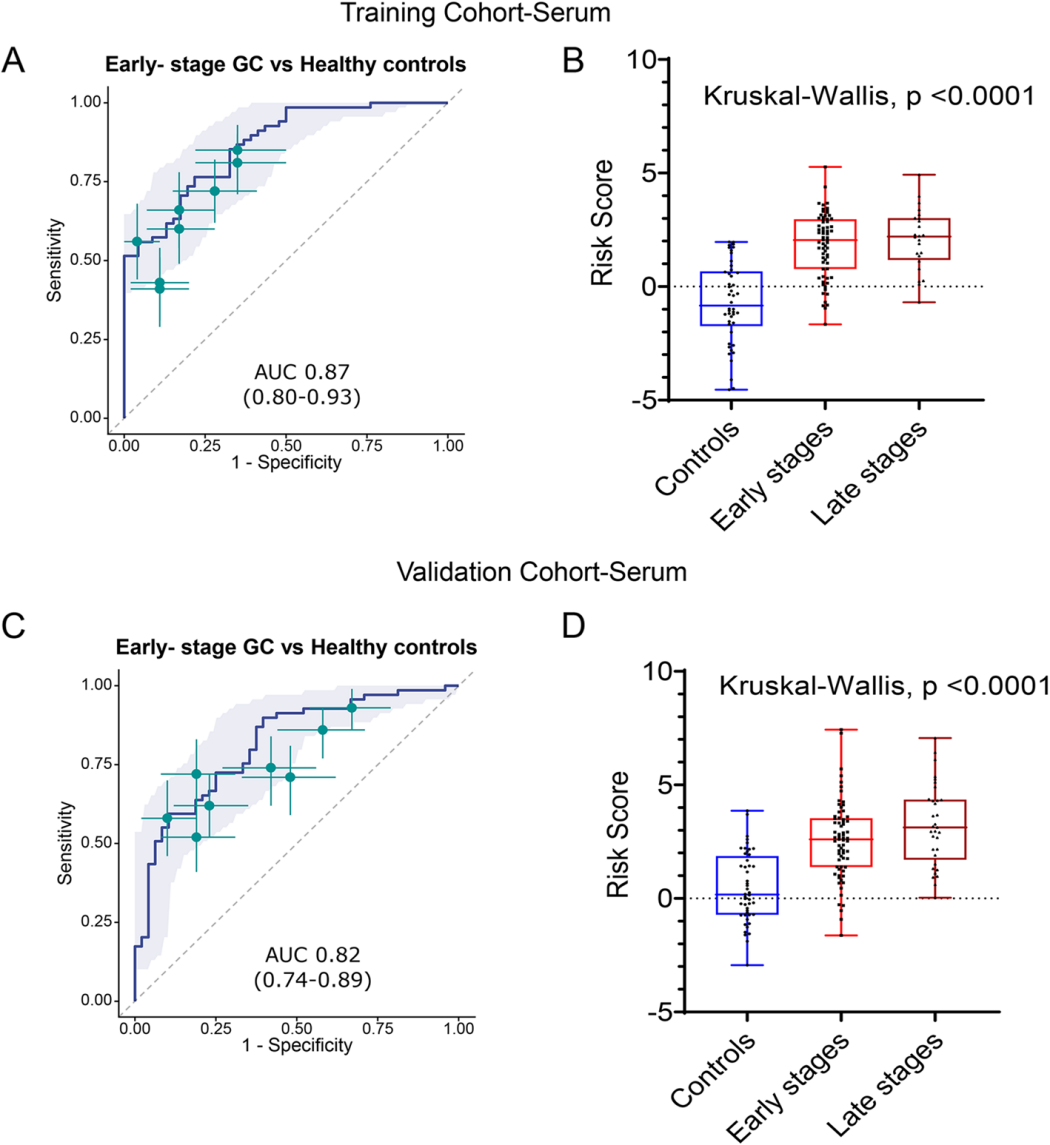
Performance of the circRNA biomarker panel to identify early stage GC patients. **A** ROC curve analysis to identify early stage GC patients from non-disease controls and **B** Risk score analysis based on risk prediction formulae in early stages (stage I and II), late stages (stage III and IV) GC patients and non-disease control subjects in serum specimens from the training cohort patients. **C** ROC curve analysis for the identification of early stage GC patients from non-disease controls and **D** risk score analysis based on risk prediction formulae in early stages (stage I and II), late stages (stage III and IV) GC patients and non-disease controls in serum validation cohort. ROC curves are shown with 95% CIs. Green lines in right panel indicate 95% CIs of sensitivity and specificity for each circRNA; green points, best threshold for sensitivity and specificity

The early detection of cancer is utmost necessary to improve the survival outcomes in patients with GC. Hence, we evaluated the performance of 8-circRNA panel in patients with early stages of disease (stage I & II) and observed that in the training cohort of patients, these markers exhibited an AUC of 0.87 (95% CI 0.80–0.93), Sensitivity 0.77, Specificity 0.78 (Fig. 3A and Table 1). These results were subsequently confirmed in the validation cohort, where the performance of the biomarker panel was quite comparable with an AUC of 0.82 (95% CI 0.74–0.89), Sensitivity 0.89, Specificity 0.6 (Fig. 3C and Table 1). Furthermore, we calculated the risk scores derived from the logistic regression model and compared these in early (stage I & II) vs. late stages (stages III & IV) relative to the non-disease control subjects. It was quite reassuring to observe that our biomarker panel successfully identified patients even with the earliest stages of disease in the serum specimens obtained from the GC patients in the training (Median Risk score in Controls − 0.7068, Early stage 2.04 Late stage 2.20, Kruskal-Wallis test *p* < 0.0001; Fig. 3B) as well as in independent validation cohorts (Median Risk score in Controls 0.17, Early stage 2.60, Late stage 3.12, Kruskal-Wallis test *p* < 0.0001; Fig. 3). These results highlight that our 8-circRNA panel is not only optimal for identifying patients with all stages of GC but is equally robust in the identification of patients with the earliest disease stages.

### The circRNA panel successfully identified patients with gastric cancer irrespective of tumor histology

**Fig. 4:**
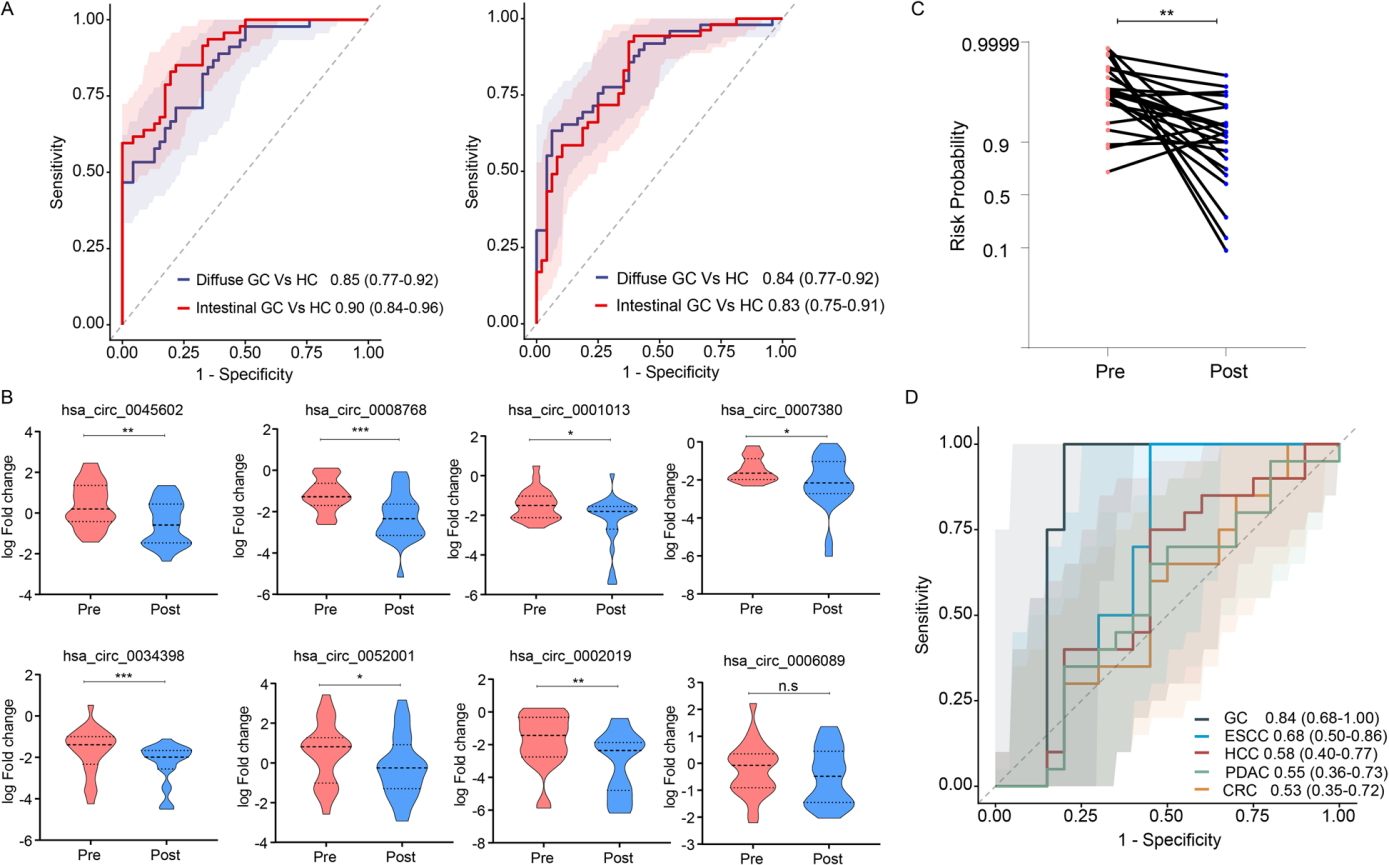
Evaluation of the circRNA panel based on tumor histology, pre vs. post-surgery specimens and their expression in GC vs. other gastrointestinal cancers. **A** The diagnostic performance of biomarker panel according to tumor histology (diffuse and intestinal type) in both serum training and validation cohorts. **B** Expression of candidate circRNAs in pre- and post-surgery serum specimens from a prospective cohort of GC patients. **C** Assessment of risk probability based on risk prediction formula between pre-and post-surgery GC specimens. **D** Performance of liquid biopsy based circRNAs biomarker panel across different GI malignancies (gastric cancer, GC; colorectal cancer, CRC; pancreatic ductal adenocarcinoma, PDAC; esophageal squamous cell carcinoma, ESCC; hepatocellular cancer, HCC)

Next, we questioned whether our biomarker panel might perform better in specific subgroups of GC patients based upon their tumor histology, diffuse vs. intestinal. It was interesting to note that our biomarker panel performed comparably for the identification of diffuse GC relative to controls with an AUC of 0.85 (95% CI 0.77–0.92), Sensitivity 0.89, Specificity 0.61 and intestinal GC relative to controls with an AUC of 0.90; (95% CI 0.84–0.96), Sensitivity 0.83, Specificity 0.8 in the training cohort (Fig. 4A; left panel). These results were subsequently validated in the serum specimens from the independent validation cohort, where this panel was equally impressive in the identification of diffuse GC (AUC = 0.84; 95% CI 0.77–0.92, Sensitivity 0.63, Specificity 0.94) and intestinal (AUC = 0.83; 95% CI 0.77–0.92, Sensitivity 0.92, Specificity 0.62) types of gastric cancers (Fig. 4A; right panel); highlighting the ability of our biomarker panel for the diagnosis of GC across all histologic subtypes.

### The circRNA panel exhibited remarkable specificity for gastric cancer compared to other gastrointestinal cancers

To further evaluate the specificity of our 8-circRNA panel, we used a two-pronged approach. First, we evaluated its performance in a prospective cohort of serum specimens obtained from a subset of patients where we obtained a baseline serum sample (pre-surgery) and a follow-up specimen 3 months after curative surgery (post-surgery). It was interesting to note that 7 of 8 circRNA markers exhibited a statistically significant decrease in expression in post-surgery serum specimens (Fig. 4B). Furthermore, we calculated the risk probability in this subset of patients and observed an overall significant reduced risk probability in post-surgery serum specimens (*p* < 0.01; Fig. 4C). These data are quite encouraging as they reinforce the specificity of our biomarkers for GC, wherein once the gastric cancer was surgically resected, the levels of these biomarkers significantly decreased in systemic circulation in the post-surgery serum specimens.

In the second approach, we evaluated the diagnostic performance of our 8-marker panel in the serum specimens from not only the patients with GC, but also other gastrointestinal (GI) cancers including colorectal cancer (CRC), pancreatic ductal adenocarcinoma (PDAC), esophageal squamous cell carcinoma (ESCC), hepatocellular cancer (HCC) and non-disease controls. It was fascinating to observe that our 8-circRNA panel exhibited a significantly higher diagnostic accuracy for the identification of patients with GC (AUC = 0.84) vs. all other GI cancers (CRC, 0.53; ESCC, 0.68; HCC, 0.58; and PDAC, 0.55; Fig. 4D). The DeLong’s test also revealed that the circRNA panel was highly specific for GC diagnosis vs. other GI cancers (compared with GC, *p* = 0.0016 for CRC, *p* = 0.011 for ESCC, *p* = 0.0010 for HCC and *p* = 0.0010 for PDAC). Collectively, these results indicate that our 8-circRNA biomarker panel is highly specific for the identification of patients with GC vs. other GI malignancies, even as a blood-based, non-invasive assay.

## Discussion

Despite recent advances in the treatment strategies, the mortality rates associated with GC still remain quite high, primarily because most of the patients with this disease are diagnosed at very advanced stages when the curative options are limited [28]. Although endoscopic surveillance has been established in many East Asian countries for GC screening, due to its invasive nature, high cost, patient discomfort, and lower incidence rates, their adaptation as GC screening approaches globally has been challenging [3, 4]. Nonetheless, given the high degree of morbidity and mortality rates associated with GC worldwide, there is an urgent need to possibly develop rapid, inexpensive and non-invasive approaches for the early detection of patients with GC. While various transcriptomic biomarkers have been pursued over the past decade in this regard, recent evidence indicates that compared to mRNAs and miRNAs, circRNAs are far more abundant, less prone to degradation due to their circular configuration, and are easily detectable in tissues, blood, and other body fluids – making them ideal candidates as non-invasive biomarkers [16]. Accordingly, in the present study, we undertook a comprehensive and systematic biomarker discovery and validation approach to develop circRNAs as liquid biopsy-based biomarkers for the early detection of GC.

We performed biomarker discovery by analyzing expression profiling datasets, which led us to identify 10 circRNAs which were significantly upregulated in patients with GC vs. matched adjacent normal mucosa tissues. Next, we translated these tissue-based markers into a liquid biopsy assay in blood and evaluated their performance in multiple, independent, serum-based clinical cohorts of patients with GC and non-disease control subjects. After rigorous training and validation of markers in serum, we established an 8-circRNA biomarker panel that robustly identified patients with GC, including those with early stage of tumors (stage I & II), as well as regardless of their histological subtype (diffuse and intestinal).

To demonstrate the specificity of our markers for GC, we observed a significant decrease in expression of circRNA biomarkers in serum specimens that were collected after 3 months of curative surgery vs. pre-surgery specimens. Importantly, the expression of these markers were also associated with reduced risk probability among the GC patients post-surgery. Together, these results highlighted that once the tumors were surgically resected, this led to the decline in the expression of these biomarkers in systemic circulation in post-surgery specimens. Furthermore, we demonstrate that our circRNA panel exhibited highest diagnostic potential in patients with GC vs. other GI malignancies, highlighting the robustness of our biomarker panel for its clinical application for the early detection of patients with GC.

With regards to the functional relevance of the circRNAs in our biomarker panel, we observed that these circRNAs serve as transcripts of several key genes associated with pathogenesis in cancer. The details of the differentially expressed circRNAs and the corresponding genes, their genomic length, spliced sequence length, RNA binding protein sites and function are presented in more detail in Supplementary Table 8. We noted that most of the circRNAs (except hsa_circ_0052001) exhibited active interaction with RNA binding protein EIF4A3. As shown in the literature, EIF4A3 is a DEAD box protein, is characterized by the conserved motif Asp-Glu-Ala-Asp (DEAD), and is reported to act as a putative RNA helicase [29]. This protein has been implicated in several cellular processes involving alteration of RNA secondary structure, such as translation initiation, nuclear and mitochondrial splicing, and ribosome and spliceosome assembly. Previous studies have also reported strong associations of EIF4A3 with different cell cycle regulatory genes (CDK1 and CDK2), tumor-associated transcription factors, chemokine signaling pathways and spliceosome signaling pathways [30–33]. Furthermore, we performed literature mining to enlist the biological function of these circRNAs based on existing literature and observed that majority of these circRNAs were reported to be associated with cell proliferation, migration and invasion in GC [34–38].

In addition, we would like to acknowledge some of the limitations of our study. First, we used microarray-based datasets to discover candidate circRNAs, since there is a limited availability of sequencing datasets for GC. Second, we prioritized circRNAs that were overexpressed in tissue specimens of GC patients and further translated these in serum specimens for developing the liquid-biopsy assay for GC. However, recent reports have also suggested that downregulation of certain circRNAs, such as circYAP1 and circFAT1(e2), were also associated with poor prognosis in GC [39, 40]. Third, our study primarily included patient specimens from Asian cohorts; hence a multinational study with larger sample size might be required to further evaluate the performance of biomarkers prior to their translation into routine clinical practice.

## Conclusion

In conclusion, our study provides a novel and promising evidence for the significance of circRNA-based biomarkers for their clinical application as non-invasive, high-throughput and inexpensive liquid biopsy biomarkers for the early detection of patients with GC, especially in countries where endoscopic surveillance is not a routine screening modality for this malignancy.

## Supplementary Information

**Additional file 1: Supplementary Figure 1.** Study design for the identification of non-invasive, liquid biopsy based circRNA for early detection of GC. **Supplementary Figure 2.** (A) Decision curve analysis and (B) calibration plot analysis of candidate circRNAs based biomarker panel.

**Additional file 2: Supplementary Table 1.** Clinicopathologic characteristics of Gastric Cancer (GC) patients in a pilot tissue validation cohort. **Supplementary Table 2.** Clinicopathologic characteristics of serum training and validation cohorts. **Supplementary Table 3.** List of divergent primers designed against candidate circRNAs in this study. **Supplementary Table 4.** Summary of diagnostic performance of individual circRNAs in tissue validation cohort. **Supplementary Table 5.** Summary of diagnostic performance of individual circRNAs in serum training cohort. **Supplementary Table 6.** Univariate analysis of individual circRNA candidates in serum training cohort. **Supplementary Table 7.** Summary of diagnostic performance of individual circRNAs in serum validation cohort. **Supplementary Table 8.** Details of candidate circRNAs discovered and validated in this study.

## Data Availability

All data derived from public database are available from these sites.

## Abbreviations

ANM: Adjacent normal mucosa
AUC: Area under the curve
CA: Carbohydrate antigen
CEA: Carcinoembryonic antigen
circRNA: Circular RNA
CRC: Colorectal cancer
CI: Confidence interval
DCA: Decision curve analysis
ESCC: Esophageal squamous cell carcinoma
GC: Gastric Cancer
GEO: Gene Expression Omnibus
GI: Gastrointestinal
HCC: Hepatocellular cancer
miRNA: Micro RNA
mRNA: Messenger RNA
NPV: Negative predictive value
PCA: Principal Component Analysis
PDAC: Pancreatic ductal adenocarcinoma
PPV: Positive predictive value
RT-qPCR: Real-time quantitative reverse transcription polymerase chain reaction
ROC: Receiver operating characteristic curve

## References

[1] Sung H, Ferlay J, Siegel RL, Laversanne M, Soerjomataram I, Jemal A, Bray F (2021). Global cancer statistics 2020: GLOBOCAN estimates of incidence and mortality worldwide for 36 cancers in 185 countries. CA Cancer J Clin.

[2] Jim MA, Pinheiro PS, Carreira H, Espey DK, Wiggins CL, Weir HK (2017). Stomach cancer survival in the United States by race and stage (2001-2009): findings from the CONCORD-2 study. Cancer.

[3] Gupta N, Bansal A, Wani SB, Gaddam S, Rastogi A, Sharma P (2011). Endoscopy for upper GI cancer screening in the general population: a cost-utility analysis. Gastrointest Endosc.

[4] Levy I, Gralnek IM (2016). Complications of diagnostic colonoscopy, upper endoscopy, and enteroscopy. Best Pract Res Clin Gastroenterol.

[5] Information Committee of Korean Gastric Cancer A (2016). Korean Gastric Cancer Association nationwide survey on gastric cancer in 2014. J Gastric Cancer.

[6] Katai H, Ishikawa T, Akazawa K, Isobe Y, Miyashiro I, Oda I, Tsujitani S, Ono H, Tanabe S, Fukagawa T (2018). Five-year survival analysis of surgically resected gastric cancer cases in Japan: a retrospective analysis of more than 100,000 patients from the nationwide registry of the Japanese Gastric Cancer Association (2001-2007). Gastric Cancer.

[7] Konishi H, Ichikawa D, Komatsu S, Shiozaki A, Tsujiura M, Takeshita H, Morimura R, Nagata H, Arita T, Kawaguchi T (2012). Detection of gastric cancer-associated microRNAs on microRNA microarray comparing pre- and post-operative plasma. Br J Cancer.

[8] Liu R, Zhang C, Hu Z, Li G, Wang C, Yang C, Huang D, Chen X, Zhang H, Zhuang R (2011). A five-microRNA signature identified from genome-wide serum microRNA expression profiling serves as a fingerprint for gastric cancer diagnosis. Eur J Cancer.

[9] Shiotani A, Murao T, Kimura Y, Matsumoto H, Kamada T, Kusunoki H, Inoue K, Uedo N, Iishi H, Haruma K (2013). Identification of serum miRNAs as novel non-invasive biomarkers for detection of high risk for early gastric cancer. Br J Cancer.

[10] Wang X, Huang CJ, Yu GZ, Wang JJ, Wang R, Li YM, Wu Q (2013). Expression of group IIA phospholipase A2 is an independent predictor of favorable outcome for patients with gastric cancer. Hum Pathol.

[11] Zhou X, Yin C, Dang Y, Ye F, Zhang G (2015). Identification of the long non-coding RNA H19 in plasma as a novel biomarker for diagnosis of gastric cancer. Sci Rep.

[12] Zhu C, Ren C, Han J, Ding Y, Du J, Dai N, Dai J, Ma H, Hu Z, Shen H (2014). A five-microRNA panel in plasma was identified as potential biomarker for early detection of gastric cancer. Br J Cancer.

[13] Jung G, Hernandez-Illan E, Moreira L, Balaguer F, Goel A (2020). Epigenetics of colorectal cancer: biomarker and therapeutic potential. Nat Rev Gastroenterol Hepatol.

[14] Vedeld HM, Goel A, Lind GE (2018). Epigenetic biomarkers in gastrointestinal cancers: the current state and clinical perspectives. Semin Cancer Biol.

[15] Lorzadeh A, Romero-Wolf M, Goel A, Jadhav U (2021). Epigenetic regulation of intestinal stem cells and disease: a balancing act of DNA and histone methylation. Gastroenterology.

[16] Li S, Han L (2019). Circular RNAs as promising biomarkers in cancer: detection, function, and beyond. Genome Med.

[17] Memczak S, Jens M, Elefsinioti A, Torti F, Krueger J, Rybak A, Maier L, Mackowiak SD, Gregersen LH, Munschauer M (2013). Circular RNAs are a large class of animal RNAs with regulatory potency. Nature.

[18] Li J, Sun D, Pu W, Wang J, Peng Y (2020). Circular RNAs in cancer: biogenesis, function, and clinical significance. Trends Cancer.

[19] Panda AC (2018). Circular RNAs act as miRNA sponges. Adv Exp Med Biol.

[20] Li P, Chen S, Chen H, Mo X, Li T, Shao Y, Xiao B, Guo J (2015). Using circular RNA as a novel type of biomarker in the screening of gastric cancer. Clin Chim Acta.

[21] Ma C, Wang X, Yang F, Zang Y, Liu J, Wang X, Xu X, Li W, Jia J, Liu Z (2020). Circular RNA hsa_circ_0004872 inhibits gastric cancer progression via the miR-224/Smad4/ADAR1 successive regulatory circuit. Mol Cancer.

[22] Wang Y, Li Z, Xu S, Guo J (2020). Novel potential tumor biomarkers: circular RNAs and exosomal circular RNAs in gastrointestinal malignancies. J Clin Lab Anal.

[23] Xia S, Feng J, Lei L, Hu J, Xia L, Wang J, Xiang Y, Liu L, Zhong S, Han L, He C (2016). Comprehensive characterization of tissue-specific circular RNAs in the human and mouse genomes. Brief Bioinform.

[24] Lu R, Shao Y, Ye G, Xiao B, Guo J (2017). Low expression of hsa_circ_0006633 in human gastric cancer and its clinical significances. Tumour Biol.

[25] Ritchie ME, Phipson B, Wu D, Hu Y, Law CW, Shi W, Smyth GK (2015). limma powers differential expression analyses for RNA-sequencing and microarray studies. Nucleic Acids Res.

[26] Dudekula DB, Panda AC, Grammatikakis I, De S, Abdelmohsen K, Gorospe M (2016). CircInteractome: a web tool for exploring circular RNAs and their interacting proteins and microRNAs. RNA Biol.

[27] Nishiwada S, Sho M, Banwait JK, Yamamura K, Akahori T, Nakamura K, Baba H, Goel A (2020). A MicroRNA signature identifies pancreatic ductal adenocarcinoma patients at risk for lymph node metastases. Gastroenterology.

[28] Rawla P, Barsouk A (2019). Epidemiology of gastric cancer: global trends, risk factors and prevention. Prz Gastroenterol.

[29] Zhu Y, Ren C, Yang L (2021). Effect of eukaryotic translation initiation factor 4A3 in malignant tumors (review). Oncol Lett.

[30] Michelle L, Cloutier A, Toutant J, Shkreta L, Thibault P, Durand M, Garneau D, Gendron D, Lapointe E, Couture S (2012). Proteins associated with the exon junction complex also control the alternative splicing of apoptotic regulators. Mol Cell Biol.

[31] Ryu I, Won YS, Ha H, Kim E, Park Y, Kim MK, Kwon DH, Choe J, Song HK, Jung H, Kim YK (2019). eIF4A3 phosphorylation by CDKs affects NMD during the cell cycle. Cell Rep.

[32] Mazloomian A, Araki S, Ohori M, El-Naggar AM, Yap D, Bashashati A, Nakao S, Sorensen PH, Nakanishi A, Shah S, Aparicio S (2019). Pharmacological systems analysis defines EIF4A3 functions in cell-cycle and RNA stress granule formation. Commun Biol.

[33] Zheng X, Huang M, Xing L, Yang R, Wang X, Jiang R, Zhang L, Chen J (2020). The circRNA circSEPT9 mediated by E2F1 and EIF4A3 facilitates the carcinogenesis and development of triple-negative breast cancer. Mol Cancer.

[34] Ding L, Zhao Y, Dang S, Wang Y, Li X, Yu X, Li Z, Wei J, Liu M, Li G (2019). Circular RNA circ-DONSON facilitates gastric cancer growth and invasion via NURF complex dependent activation of transcription factor SOX4. Mol Cancer.

[35] Liu Y, Xu Y, Xiao F, Zhang J, Wang Y, Yao Y, Yang J (2020). Comprehensive analysis of a circRNA-miRNA-mRNA network to reveal potential inflammation-related targets for gastric adenocarcinoma. Mediat Inflamm.

[36] Tian Y, Xing Y, Zhang Z, Peng R, Zhang L, Sun Y (2020). Bioinformatics analysis of key genes and circRNA-miRNA-mRNA regulatory network in gastric cancer. Biomed Res Int.

[37] Zhang Y, Wang M, Zang X, Mao Z, Chen Y, Mao F, Qian H, Xu W, Zhang X (2020). CircHN1 affects cell proliferation and migration in gastric cancer. J Clin Lab Anal.

[38] Chen B, Ji F, Wen X, Jin Z (2020). Circular RNA circ_ASAP2 promotes cell viability, migration, and invasion of gastric cancer cells by regulating the miR-770-5p/CDK6 axis. Int J Clin Exp Pathol.

[39] Liu H, Liu Y, Bian Z, Zhang J, Zhang R, Chen X, Huang Y, Wang Y, Zhu J (2018). Circular RNA YAP1 inhibits the proliferation and invasion of gastric cancer cells by regulating the miR-367-5p/p27 (Kip1) axis. Mol Cancer.

[40] Fang J, Hong H, Xue X, Zhu X, Jiang L, Qin M, Liang H, Gao L (2019). A novel circular RNA, circFAT1(e2), inhibits gastric cancer progression by targeting miR-548g in the cytoplasm and interacting with YBX1 in the nucleus. Cancer Lett.

