# Supplementary figures and images for: Diagnostic efficacy of circular RNAs as noninvasive, liquid biopsy biomarkers for early detection of gastric cancer
Source: Mol Cancer. 2022 Feb 9;21:42. doi: 10.1186/s12943-022-01527-7 (PMC8826675; doi:10.1186/s12943-022-01527-7)

SUPPLEMENTARY FIGURE 1

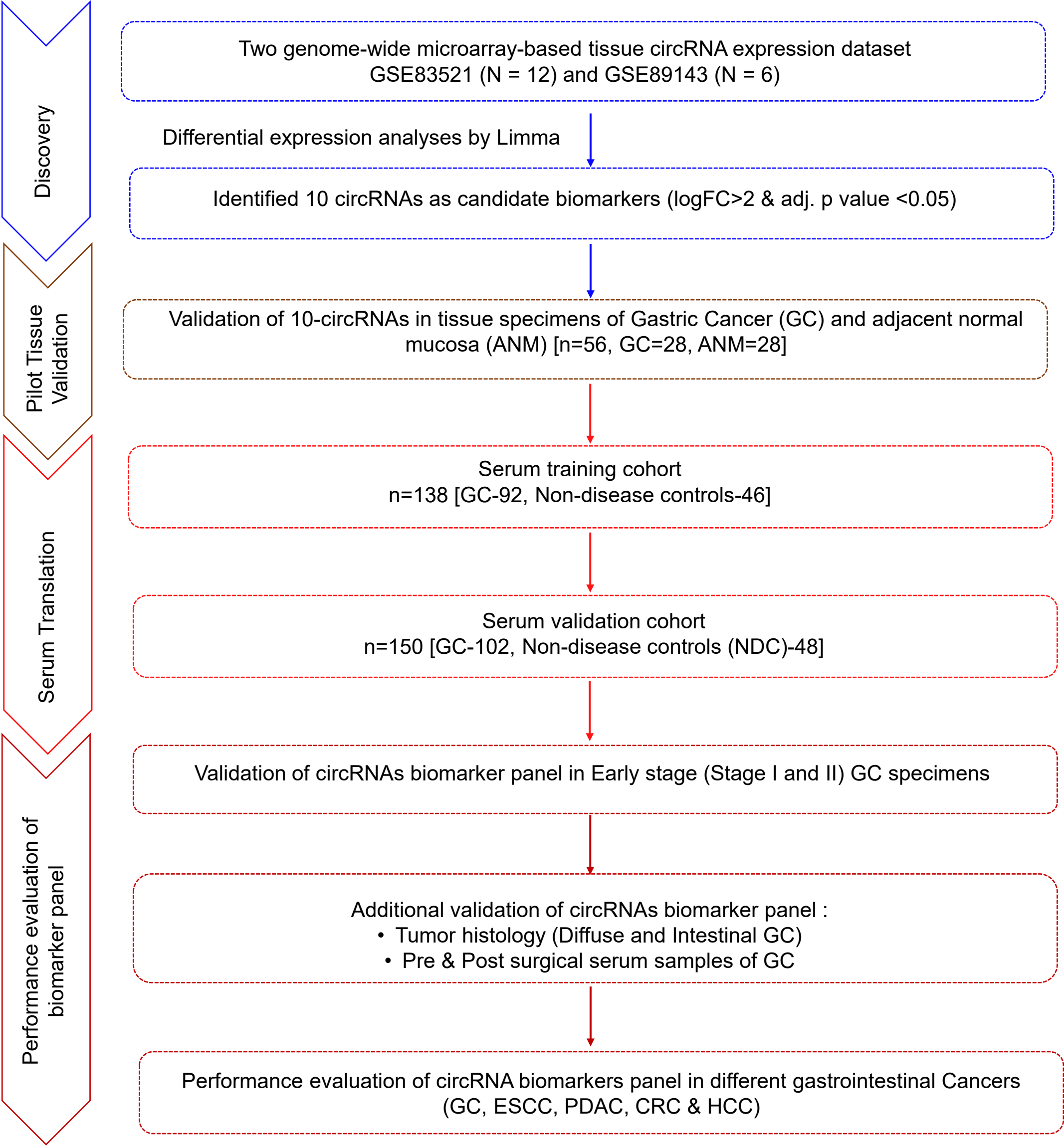

SUPPLEMENTARY FIGURE 2

A

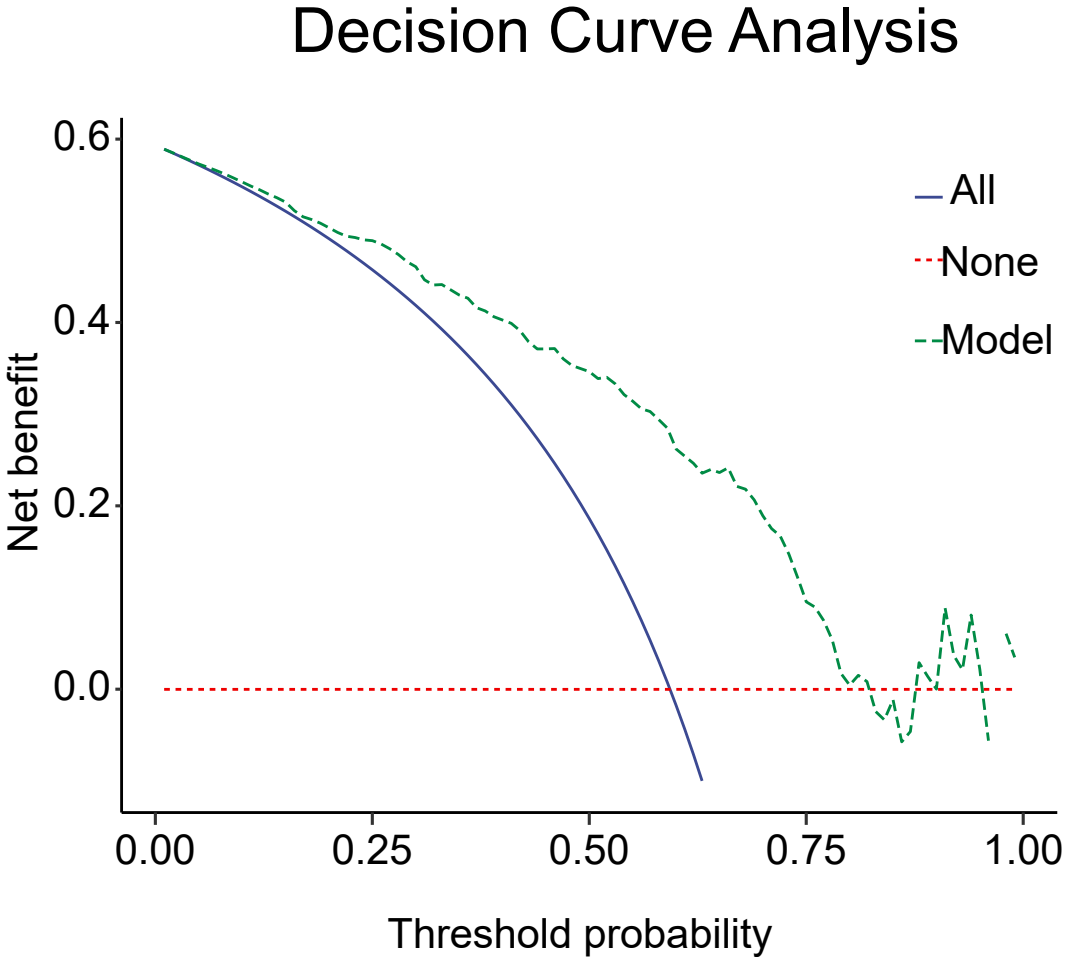

B

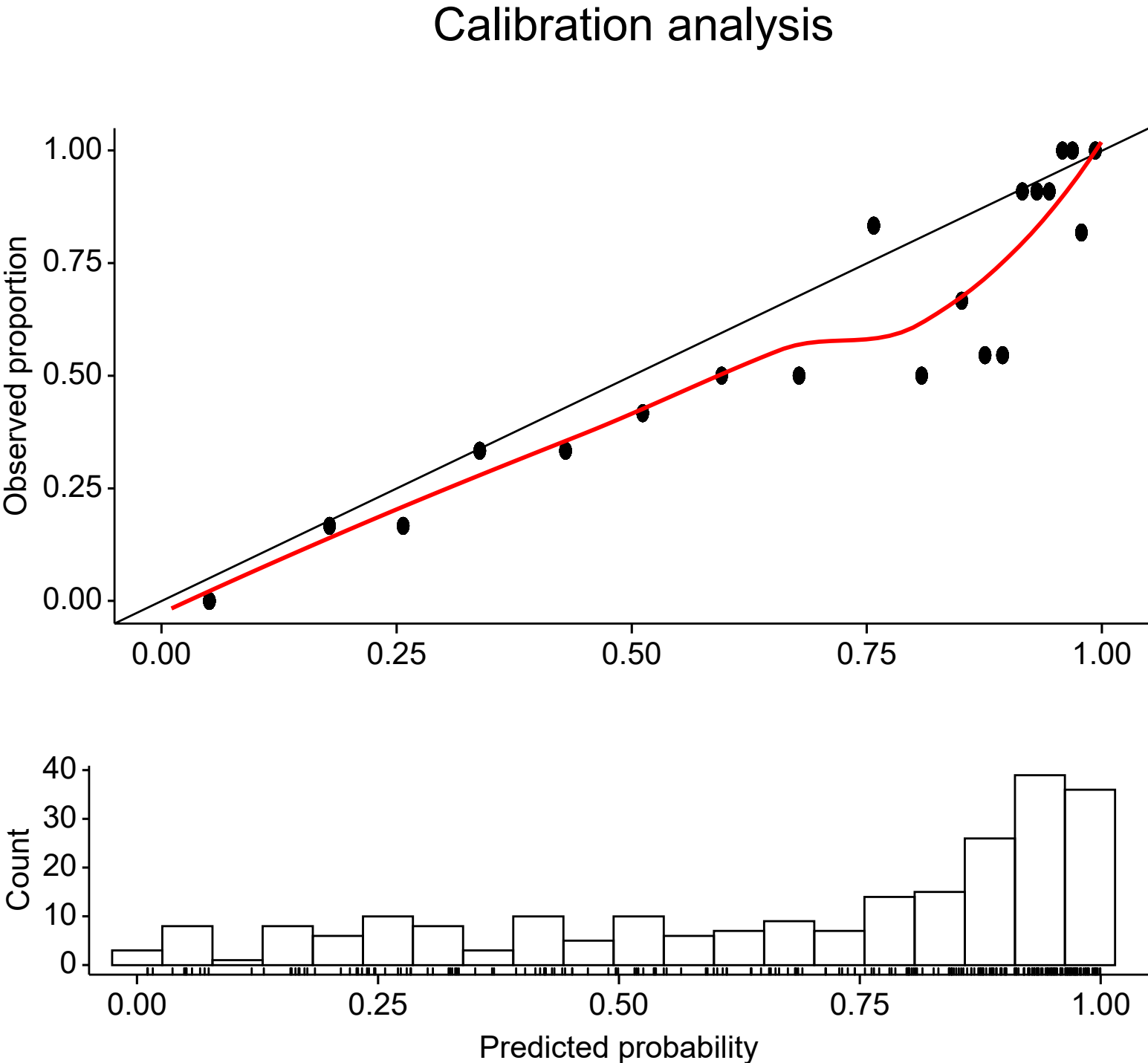

Supplement: Supplementary file 1 — Additional file 1: Supplementary Figure 1. Study design for the identification of non-invasive, liquid biopsy based circRNA for early detection of GC. Supplementary Figure 2. (A) Decision curve analysis and (B) calibration plot analysis of candidate circRNAs based biomarker panel. [file 12943_2022_1527_MOESM1_ESM.pdf]
