# Supplementary material for: Diagnostic efficacy of circular RNAs as noninvasive, liquid biopsy biomarkers for early detection of gastric cancer
Source: Mol Cancer. 2022 Feb 9;21:42. doi: 10.1186/s12943-022-01527-7 (PMC8826675; doi:10.1186/s12943-022-01527-7)
Supplement: Supplementary file 2 — Additional file 2: Supplementary Table 1. Clinicopathologic characteristics of Gastric Cancer (GC) patients in a pilot tissue validation cohort. Supplementary Table 2. Clinicopathologic characteristics of serum training and validation cohorts. Supplementary Table 3. List of divergent primers designed against candidate circRNAs in this study. Supplementary Table 4. Summary of diagnostic performance of individual circRNAs in tissue validation cohort. Supplementary Table 5. Summary of diagnostic performance of individual circRNAs in serum training cohort. Supplementary Table 6. Univariate analysis of individual circRNA candidates in serum training cohort. Supplementary Table 7. Summary of diagnostic performance of individual circRNAs in serum validation cohort. Supplementary Table 8. Details of candidate circRNAs discovered and validated in this study. [file 12943_2022_1527_MOESM2_ESM.docx]

**Supplementary Table 1.** Clinicopathologic characteristics of 28 Gastric Cancer (GC) patients in a tissue validation cohort.

| Variables | Tissue samples, n (%) |
| --- | --- |
| Median age at operation, years (range) | 68 (40-80) |
| Sex  Male  Female | 19 (67.85%)  9 (32.15%) |
| TNM stage*  1 & 2  3 & 4 | 18 (64.28%)  10 (35.72%) |
| Lauren’s classification  Diffuse  Intestinal | 10 (35.72%)  18 (64.28%) |

*TNM stage was based on AJCC 7^th^ edition

**Supplementary Table 2.** Clinicopathologic characteristics of serum training and validation cohorts.

| **Variables** | **Training cohort, n (%)** | | **Validation cohort, n (%)** | |
| --- | --- | --- | --- | --- |
|  | **Cancer patients**  **(n=92)** | **Healthy controls**  **(n=46)** | **Cancer patients**  **(n=102)** | **Healthy Controls**  **(n=48)** |
| Median age at operation, years (range) | 68 (26-92) | 64 (26-85) | 68 (39-88) | 66 (24-83) |
| Sex  Male  Female | 62 (67.4%)  30 (32.6%) | 27 (58.7%)  19 (41.3%) | 62 (60.8%)  40 (39.2%) | 19 (39.6%)  29 (60.4%) |
| TNM stage*  1 & 2  3 & 4 | 68 (73.9%)  24 (26.1%) | N. A  N. A | 69 (67.6%)  33 (32.4%) | N. A  N. A |
| Lauren’s classification  Diffuse  Intestinal | 45 (48.9%)  47 (51.1%) | N. A  N. A | 49 (48%)  53 (52%) | N. A  N. A |

*TNM stage was based on AJCC 7^th^ edition

| **circRNA ID** | **Left Primer** | **Right Primer** |
| --- | --- | --- |
| hsa_circ_0045602 | GCAGGGAAGACTTGGAGTCA | GGCCATTTTGTTCTTCCTCA |
| hsa_circ_0055521 | AGGCAAGAGAAAGACCGTGA | TCCAGTGGACAATGCAGAAA |
| hsa_circ_0034398 | GGCCAATGAGGTGGACTATG | TCCTGGGAGAAGATGCTCAG |
| hsa_circ_0052001 | CCACGTGTACGCAGTGACC | CATCTCCACAATGGCTTCTG |
| hsa_circ_0006089 | AGCTGGGGGTTCATTATTCC | TCCTCAGGGTTGGTCTTCAC |
| hsa_circ_0001013 | GATCAACGGCGAATGACTGA | AGAAGAATCGCTGGAACCCA |
| hsa_circ_0007380 | TAGCATGATCCCACCACTGC | AGTGTGGCGATTTTCTCAAGA |
| hsa_circ_0004339 | TTGCTACCTCAGGTCCACAG | GGAATATCAGGCTCGGAGAA |
| hsa_circ_0008768 | GGGAATGCAGGCTTTAATGA | TCCTCAGGGTTGGTCTTCAC |
| hsa_circ_0002019 | GCTGGCTTCAGAAAAGGATG | TGAAAAGAGGGAGAGCTGGA |
| ß-actin | TCACCCACACTGTGCCCATCTACGA | CAGCGGAACCGCTCATTGCCAATGG |

**Supplementary Table 3.** List of divergent primers designed against candidate circRNAs in this study.

**Supplementary Table 4.** Summary of diagnostic performance of individual circRNAs in pilot tissue validation cohort.

|  | AUC (CI) | Accuracy (CI) | Precision (CI) | Sensitivity (CI) | Specificity (CI) | NPV (CI) |
| --- | --- | --- | --- | --- | --- | --- |
| hsa_circ_0055521 | 0.82 (0.71-0.94) | 0.79 (0.68-0.89) | 0.79 (0.67-0.92) | 0.79 (0.64-0.93) | 0.79 (0.61-0.93) | 0.79 (0.67-0.92) |
| hsa_circ_0034398 | 0.82 (0.71-0.94) | 0.80 (0.70-0.89) | 0.76 (0.66-0.88) | 0.89 (0.75-1.00) | 0.71 (0.54-0.89) | 0.88 (0.74-1.00) |
| hsa_circ_0001013 | 0.77 (0.64-0.90) | 0.77 (0.66-0.88) | 0.76 (0.65-0.88) | 0.79 (0.64-0.93) | 0.75 (0.57-0.89) | 0.78 (0.67-0.92) |
| hsa_circ_0007380 | 0.76 (0.63-0.89) | 0.77 (0.66-0.88) | 0.78 (0.66-0.91) | 0.75 (0.57-0.89) | 0.79 (0.61-0.93) | 0.76 (0.65-0.89) |
| hsa_circ_0004339 | 0.78 (0.65-0.91) | 0.79 (0.68-0.88) | 0.74 (0.64-0.84) | 0.89 (0.75-1.00) | 0.68 (0.50-0.82) | 0.87 (0.73-1.00) |
| hsa_circ_0008768 | 0.83 (0.72-0.94) | 0.84 (0.73-0.93) | 0.83 (0.71-0.96) | 0.86 (0.71-0.96) | 0.82 (0.68-0.96) | 0.86 (0.74-0.96) |
| hsa_circ_0045602 | 0.88 (0.78-0.97) | 0.84 (0.73-0.93) | 0.88 (0.77-1.00) | 0.79 (0.61-0.93) | 0.89 (0.79-1.00) | 0.81 (0.70-0.93) |
| hsa_circ_0006089 | 0.81 (0.69-0.93) | 0.80 (0.70-0.91) | 0.77 (0.67-0.89) | 0.86 (0.71-0.96) | 0.75 (0.57-0.89) | 0.84 (0.72-0.96) |
| hsa_circ_0052001 | 0.80 (0.68-0.92) | 0.79 (0.68-0.88) | 0.90 (0.77-1.00) | 0.64 (0.46-0.82) | 0.93 (0.82-1.00) | 0.72 (0.62-0.84) |
| hsa_circ_0002019 | 0.88 (0.79-0.97) | 0.82 (0.71-0.91) | 0.85 (0.73-0.96) | 0.79 (0.64-0.93) | 0.86 (0.71-0.96) | 0.80 (0.70-0.93) |

**Supplementary Table 5.** Summary of diagnostic performance of individual circRNAs in serum training cohort.

| circRNA | AUC (CI) | Accuracy (CI) | Precision (CI) | Sensitivity (CI) | Specificity (CI) | NPV (CI) |
| --- | --- | --- | --- | --- | --- | --- |
| hsa_circ_0052001 | 0.68 (0.58-0.77) | 0.61 (0.53-0.68) | 0.90 (0.81-0.98) | 0.47 (0.37-0.57) | 0.89 (0.78-0.98) | 0.46 (0.40-0.51) |
| hsa_circ_0001013 | 0.77 (0.69-0.86) | 0.71 (0.63-0.78) | 0.88 (0.81-0.95) | 0.65 (0.55-0.75) | 0.83 (0.70-0.93) | 0.54 (0.47-0.62) |
| hsa_circ_0007380 | 0.77 (0.70-0.85) | 0.70 (0.62-0.78) | 0.90 (0.84-0.97) | 0.62 (0.52-0.72) | 0.87 (0.76-0.96) | 0.53 (0.47-0.61) |
| hsa_circ_0045602 | 0.81 (0.74-0.89) | 0.79 (0.72-0.86) | 0.85 (0.79-0.91) | 0.84 (0.75-0.90) | 0.70 (0.57-0.83) | 0.68 (0.57-0.79) |
| hsa_circ_0008768 | 0.77 (0.69-0.85) | 0.75 (0.69-0.82) | 0.80 (0.75-0.86) | 0.84 (0.76-0.90) | 0.59 (0.43-0.72) | 0.64 (0.53-0.76) |
| hsa_circ_0034398 | 0.64 (0.55-0.74) | 0.59 (0.51-0.67) | 0.84 (0.75-0.93) | 0.47 (0.37-0.57) | 0.83 (0.72-0.91) | 0.44 (0.38-0.50) |
| hsa_circ_0006089 | 0.69 (0.60-0.78) | 0.66 (0.58-0.73) | 0.87 (0.79-0.94) | 0.58 (0.47-0.67) | 0.83 (0.70-0.91) | 0.49 (0.43-0.57) |
| hsa_circ_0002019 | 0.78 (0.71-0.86) | 0.75 (0.68-0.82) | 0.82 (0.77-0.88) | 0.80 (0.72-0.88) | 0.65 (0.50-0.78) | 0.63 (0.52-0.74) |

**Supplementary Table 6.** Univariate analysis for circRNA candidates in serum training cohort.

| circRNA | OR | 95% CI | Estimate | *P* |
| --- | --- | --- | --- | --- |
| hsa_circ_0052001 | 1.619 | 1.13-2.383 | 0.482 | 0.011 |
| hsa_circ_0001013 | 3.309 | 2.034-5.837 | 1.197 | < 0.001 |
| hsa_circ_0007380 | 2.248 | 1.508-3.556 | 0.81 | < 0.001 |
| hsa_circ_0045602 | 1.58 | 1.047-2.958 | 0.458 | 0.077 |
| hsa_circ_0008768 | 2.578 | 1.684-4.234 | 0.947 | < 0.001 |
| hsa_circ_0034398 | 1.358 | 0.954-1.952 | 0.306 | 0.091 |
| hsa_circ_0006089 | 1.688 | 1.172-2.486 | 0.523 | 0.006 |
| hsa_circ_0002019 | 3.192 | 2.048-5.27 | 1.161 | < 0.001 |

**Supplementary Table 7.** Summary of diagnostic performance of individual circRNAs in serum validation cohort.

| circRNA | AUC (CI) | Accuracy (CI) | Precision (CI) | Sensitivity (CI) | Specificity (CI) | NPV (CI) |
| --- | --- | --- | --- | --- | --- | --- |
| hsa_circ_0045602 | 0.77 (0.68-0.86) | 0.76 (0.69-0.82) | 0.89 (0.83-0.95) | 0.74 (0.65-0.82) | 0.81 (0.69-0.92) | 0.59 (0.51-0.68) |
| hsa_circ_0008768 | 0.62 (0.53-0.72) | 0.65 (0.58-0.73) | 0.76 (0.70-0.82) | 0.72 (0.64-0.80) | 0.52 (0.38-0.67) | 0.46 (0.36-0.57) |
| hsa_circ_0006089 | 0.70 (0.62-0.79) | 0.74 (0.68-0.80) | 0.77 (0.72-0.81) | 0.89 (0.83-0.95) | 0.42 (0.27-0.56) | 0.65 (0.50-0.79) |
| hsa_circ_0052001 | 0.71 (0.63-0.80) | 0.64 (0.57-0.71) | 0.92 (0.85-0.98) | 0.52 (0.42-0.61) | 0.90 (0.81-0.98) | 0.47 (0.42-0.52) |
| hsa_circ_0034398 | 0.64 (0.54-0.73) | 0.73 (0.67-0.78) | 0.74 (0.71-0.78) | 0.91 (0.85-0.96) | 0.33 (0.21-0.46) | 0.64 (0.46-0.81) |
| hsa_circ_0002019 | 0.71 (0.62-0.80) | 0.69 (0.63-0.77) | 0.79 (0.74-0.85) | 0.75 (0.66-0.82) | 0.58 (0.44-0.73) | 0.52 (0.42-0.62) |
| hsa_circ_0007380 | 0.76 (0.68-0.84) | 0.67 (0.59-0.74) | 0.88 (0.81-0.95) | 0.59 (0.49-0.69) | 0.83 (0.73-0.94) | 0.49 (0.43-0.56) |
| hsa_circ_0001013 | 0.70 (0.62-0.79) | 0.63 (0.55-0.70) | 0.86 (0.79-0.93) | 0.54 (0.45-0.64) | 0.81 (0.69-0.92) | 0.45 (0.40-0.52) |

**Supplementary Table 8.** Details of candidate circRNAs discovered and validated in this study.

| circRNAs ID | Location | Associated Genes | Genomic Length (bp) | Spliced Seq Length (bp) | RNA binding protein sites matching to circRNAs | RNA-binding protein sites matching flanking regions of circRNA | Function | Reference |
| --- | --- | --- | --- | --- | --- | --- | --- | --- |
| hsa_circ_0006089 | chr2:9490936-9499018 | ASAP2 | 8082 | 438 | EIF4A3, FUS, HuR | EIF4A3, TDP43 | Not reported | - |
| hsa_circ_0008768 | chr2:9490936-9508648 | ASAP2 | 17712 | 533 | EIF4A3, FUS, HuR | EIF4A3, TDP43 | Promotes cell viability, migration, and invasion of gastric cancer cells | [1] |
| hsa_circ_0001013 | chr2:61339656-61345251 | KIAA1841 | 5595 | 3741 | EIF4A3, U2AF65 | EIF4A3, U2AF65 | Associated with PI3K-Akt signaling pathway and ECM-receptor interaction in GC. | [2] |
| hsa_circ_0045602 | chr17:73142760-73144766 | HN1 | 2006 | 260 | AGO1, AGO2, C22ORF28, DGCR8, EIF4A3, FMRP, HUR, IGF2BP1, IGF2BP3 | EIF4A3, HuR, U2AF65 | Associated with proliferation and migration in GC. | [3] |
| hsa_circ_0002019 | chr11:126142863-126143349 | FOXRED1 | 486 | 230 | EIF4A3, AGO2, HuR | AGO2, EIF4A3, PTB | Not reported | - |
| hsa_circ_0055521 | chr2:86433228-86434450 | MRPL35 | 1222 | 335 | EIF4A3 | EIF4A3 | Regulates the development of GC | [4] |
| hsa_circ_0034398 | chr15:36936478-36950106 | C15orf41 | 13628 | 245 | AGO1, AGO2, AGO3, EIF4A3, HuR | EIF4A3 | Associated with autoimmune diseases | [5] |
| hsa_circ_0052001 | chr19:50720871-50721028 | MYH14 | 157 | 157 | - | - | Not reported |  |
| hsa_circ_0007380 | chr5:6623326-6625782 | NSUN2 | 2456 | 178 | EIF4A3, AGO2, HuR, IGF2BP3 | DGCR8, EIF4A3, HNRNPC, HuR, IGF2BP2, PTB, U2AF65 | Associated with promotion of colorectal liver metastasis | [6] |
| hsa_circ_0004339 | chr21:34953607-34958487 | DONSON | 4880 | 948 | AGO1, AGO2, C22ORF28, CAPRIN1, EIF4A3, FMRP, HUR, IGF2BP1, IGF2BP2 | EIF4A3, HuR, U2AF65 | Associated with growth and invasion of GC | [7] |

**References:**

1. Chen B, Ji F, Wen X, Jin Z: Circular RNA circ_ASAP2 promotes cell viability, migration, and invasion of gastric cancer cells by regulating the miR-770-5p/CDK6 axis. International journal of clinical and experimental pathology 2020, 13:2806-2819.

2. Tian Y, Xing Y, Zhang Z, Peng R, Zhang L, Sun Y: Bioinformatics Analysis of Key Genes and circRNA-miRNA-mRNA Regulatory Network in Gastric Cancer. BioMed research international 2020, 2020:2862701-2862701.

3. Zhang Y, Wang M, Zang X, Mao Z, Chen Y, Mao F, Qian H, Xu W, Zhang X: CircHN1 affects cell proliferation and migration in gastric cancer. Journal of clinical laboratory analysis 2020, 34:e23433-e23433.

4. Liu Y, Xu Y, Xiao F, Zhang J, Wang Y, Yao Y, Yang J: Comprehensive Analysis of a circRNA-miRNA-mRNA Network to Reveal Potential Inflammation-Related Targets for Gastric Adenocarcinoma. Mediators of inflammation 2020, 2020:9435608-9435608.

5. Xia X, Tang X, Wang S: Roles of CircRNAs in Autoimmune Diseases. Frontiers in immunology 2019, 10:639-639.

6. Chen R-X, Chen X, Xia L-P, Zhang J-X, Pan Z-Z, Ma X-D, Han K, Chen J-W, Judde J-G, Deas O, et al: N6-methyladenosine modification of circNSUN2 facilitates cytoplasmic export and stabilizes HMGA2 to promote colorectal liver metastasis. Nature Communications 2019, 10:4695.

7. Ding L, Zhao Y, Dang S, Wang Y, Li X, Yu X, Li Z, Wei J, Liu M, Li G: Circular RNA circ-DONSON facilitates gastric cancer growth and invasion via NURF complex dependent activation of transcription factor SOX4. Molecular cancer 2019, 18:45-45.
